# The predictors of COVID-19 mortality among health systems parameters: an ecological study across 203 countries

**DOI:** 10.1186/s12961-022-00878-3

**Published:** 2022-06-27

**Authors:** Sutapa Bandyopadhyay Neogi, Shivam Pandey, G. S. Preetha, Sumant Swain

**Affiliations:** 1 International Institute of Health Management Research (IIHMR), Plot 3, Sector 18A, Dwarka, New Delhi, 110 075 India; 2 Department of Biostatistics, All India Institute of Medical Science (A.I.I.M.S), New Delhi, India

**Keywords:** COVID, Health systems, Resilience, Global health security, Health expenditure

## Abstract

**Background:**

Health systems responsiveness is the key to addressing infectious disease threats such as pandemics. The paper outlines an assessment of health systems resilience by exploring the association of health systems and Global Health Security (GHS) parameters with case load and mortality resulting from COVID-19 across 203 countries using an ecological design.

**Methodology:**

Correlation analysis was performed to assess the relationship of each of the indicators with COVID 19 cases and deaths per million population. Stepwise multiple regression models were developed to determine the predictors of COVID-19 cumulative cases and deaths per million population separately.

**Results:**

Global health security indicators seemed to have a strong association when analyzed individually but those did not necessarily translate into less burden of cases or deaths in the multivariable analysis. The predictors of cumulative deaths per million population included general government expenditure on health as a proportion of general government expenditure, responsiveness of the system to prevent the emergence and release of pathogens and governance related voice and accountability.

**Conclusion:**

To conclude, health financing parameters and preventive activities with regard to emergence of pathogens were better predictors of cumulative COVID-19 cases and deaths per million population compared to other health systems and global health security indicators.

## Key messages

- Health financing parameters are better predictors of cumulative COVID-19 cases and deaths per million population compared to other health systems and global health security indicators.
- More robust and valid indicators are required to assess the performance of health system.

## Introduction

COVID-19, caused by the novel SARS-CoV-2 is the worst catastrophe in this century that affected more than 228 million people and caused more than 4.6 million deaths globally [1]. The proportion of cases was maximum in the Americas (39%), followed by Europe (30%), South-East Asia (19%), Eastern Mediterranean (7%), Western Pacific (3%), and Africa (3%) among WHO regions. Similar trend was observed regarding distribution of deaths, with Americas leading with 46% of the burden followed by Europe (28%), South-East Asia (14%), Eastern Mediterranean (6%), Western Pacific (2%), and Africa (3%) [1]. The health systems have been struggling to play a vital role to minimise the case load and morbidity by instituting preventive measures and avert mortality by providing appropriate case management.

Health system is defined as comprising all the resources, organizations, and institutions, which produce interdependent actions aimed principally at improving, maintaining, or restoring health. Health system responsiveness displays the preparedness of nations to tackle health emergencies, and not be overwhelmed by the sudden and disproportionate increase in the demand of health facilities [2]. The responsiveness of health systems is difficult to measure, yet an assessment of parameters of health systems building blocks and Global Health Security (GHS) frameworks is a significant step in the journey towards a holistic Health Systems Strengthening (HSS). The performance of health systems is gauged by assessing the parameters of its building blocks (encompassing service delivery, health workforce, medical products, health financing, health information system, leadership, and governance), within the overarching goals of better health, responsiveness to the expectations of the population, and equity of financial contribution with protection against financial risk. In order to combat any public health crises, a set of activities are required to minimize the danger and impact of acute public health events that endanger the collective health of populations living across geographical regions and international boundaries, collectively embedded under global public health security measures. The parameters of GHS cover prevention of emergence or release of pathogens, early detection and reporting for epidemics of potential international concern, rapid response to and mitigation of the spread of an epidemic, sufficient and robust health system to treat the sick and protect health workers, commitment to improving national capacity, financing and adherence to norms and overall risk environment and country vulnerability to biological threats. These parameters, too fall under the ambit of health systems, that are aimed to improve epidemic detection, preparedness, response, and case management.

A preliminary analysis was done to assess the strengths of health systems of select countries and their capacities to respond to pandemic threats using WHO and GHS frameworks, which had exposed the weaknesses of global health systems preparedness, the inability to respond timely in most countries, and the ineffectiveness of policy responses in many instances [3].

Reports have stated that a country’s health system is the first line of defence in the face of any crisis, and if the system is not resilient, it will be overwhelmed and will collapse, exacerbating the health impact and adding to inequality. Countries such as Germany, New Zealand, South Korea, Taiwan Province of China, and Vietnam have demonstrated resilience in their health systems and therefore could tackle COVID-19 better [4].

GHS index has been considered as a benchmark to assess the preparedness of countries to tackle the pandemic. Enough evidence is available on the correlation of GHS index with COVID cases and deaths. Abbey et al. analyzed data of COVID cases and deaths of 36 countries, mostly from high income group [5]. Ji et al. analyzed cases and deaths from 178 countries based on GHS index to establish the correlation before and after institution of lockdown for different countries [6]. In another analysis of 52 countries, the effect of climatic conditions and GHS on the transmission of COVID was studied [7]. Yet another analysis on 100 countries assessed the testing rate as a surrogate marker of health systems capacity [8]. A more comprehensive analysis was undertaken on health systems indicators but it was restricted to 54 countries in Africa [9].

These analyses were restricted to GHS primarily. There is a paucity of evidence on a comprehensive analysis of health systems indicators that goes beyond establishing correlation. GHS index was developed keeping pandemics at the core. Health systems resilience required deeper analysis beyond the realms of GHS. We therefore undertook an analysis on health systems resilience by exploring the association of health systems inclusive of GHS parameters with case load and mortality resulting from COVID-19 across all countries. The objective of the analysis is to identify the health systems and GHS factors that are most predictive of mortality due to COVID-19 in all countries across WHO regions.

## Materials and methods

The study followed an ecological design with country as the unit of analysis. Secondary data of 203 countries across all WHO region countries, available in the public domain were analyzed. The WHO health systems framework and GHS parameters formed the explanatory variables [10, 11]. The performance of each building block was assessed with respect to the standardized indicators proposed by the WHO [10]. A list of GHS indicators has been drawn from the existing literature [11].

For the purpose of analysis, two outcome indicators were considered that included COVID-19 cumulative cases per one million population and cumulative deaths per one million population as on 1 November 2020, 10 am CEST, for every country. The underlying assumption behind considering the cumulative number of cases is that timely initiation of measures such as isolation of patients in hospitals or home which indirectly is related to numbers of doctors, nurses and ability to make more beds available will dampen the spread of infection. A sensitive health system is bound to mobilize its resources to isolate, manage and treat COVID-19 patients efficiently to avert deaths. We therefore considered cumulative deaths as a key outcome variable.

The data on cumulative cases and deaths were obtained from WHO-Weekly epidemiological update [1]. The purpose of this restriction was to have data that were not affected by immunization. We presumed that population level vaccination drive was initiated in phases after October 2020 in different parts of the world.

Data for rest of the variables were the latest ones as available on 1 November 2020.

Box 1: List of health systems indicators used in the study as explanatory variables ***Health service delivery***

- Number of inpatient beds per 10,000 population

***Health workforce***

- Number of health workers per 10,000 population
- Annual number of graduates of health professions educational institutions per 100,000 population
  o Doctors
  p Nurses
- Doctor population ratio (per 10,000 population)
- Nursing and midwifery personnel (per 1000 population)

***Health information system***

- Civil registration coverage of births (%)
- Civil registration coverage of cause of death (%)
- Ill-defined causes in cause-of-death registration (%)

***Essential medicines***

- Median availability of selected generic medicines in public health facilities
- Median availability of selected generic medicines in private health facilities

***Health financing***

- Current health expenditure (as % of GDP)
- Domestic general government health expenditure as % of GDP (2017)
- General government expenditure on health as a proportion of general government expenditure
- Out-of-pocket payments for health (% of current expenditure on health)

***Governance***

- Voice and accountability (− 2.5 to 2.5)
- Political stability (− 2.5 to 2.5)
- Government effectiveness (− 2.5 to 2.5)
- Regulatory quality (− 2.5 to 2.5)
- Rule of law (− 2.5 to 2.5)
- Control of corruption (− 2.5 to 2.5)

*Global health security indicators: (all out of 100)*

- Prevention of emergence or release of pathogens
- Early detection and reporting for epidemics of potential international concern
- Rapid response to and mitigation of the spread of an epidemic
- Sufficient and robust health system to treat the sick and protect health workers
- Commitment to improving national capacity, financing and adherence to norms
- Overall risk environment and country vulnerability to biological threats

***Other indicators (%)***

- Female literacy rate
- Proportion of people aged 60 years or more
- Proportion of people below poverty line

### Statistical analysis

The analysis was performed by considering all countries combined. It focused on exploring the predictors of COVID-19 cumulative cases and deaths per million population. Descriptive characteristics of the health system factors for all the countries have been presented. Correlation analysis was performed using Pearson’s correlation, and Spearman’s rank correlation, as appropriate. Subsequently, stepwise multiple linear regression models were developed to determine the predictors of COVID-19 cumulative cases and deaths per million population separately. Variables that had a strong correlation in bivariate analysis, had about 80% data available and seemed to be a plausible explanation for COVID-19 infection and deaths were considered in the models. All analyses were performed in STATA version 16 (StataCorp, College Station,Texas) and SPSS version 21.

## Results

Data compiled from WHO repository reflected incompleteness of data for several countries. Missing data were more for indicators related to health workforce and essential medicines. These missing variables were from different regions except South East Asia. (Table 1).

**Table 1:** Descriptive characteristics of health systems factors for countries

Variable	*N*	Mean value	Min	Max	Std. dev
*Health service delivery*					
Number of inpatient beds per 10,000 population^a^	159	26.25	1	143	23.98
*Health workforce*					
Number of health workers per 10,000 population^a^	34	54.39	5.7	151.9	45.89
Annual number of graduates (doctors) of health professions educational institutions per 100,000 population^b^	36	14.61	3.3	56	8.73
Annual number of graduates (nurses) of health professions educational institutions per 100,000 population^b^	34	45.02	14	101	24.88
Doctor population ratio (per 10,000 pop)^c^	185	17.78	0.008	85.5	17.15
Nursing and midwifery personnel (per 10,000 pop)^d^	185	42.75	0.6	201.609	43.19
*Health information*					
Civil registration coverage of births (%)	170	84.16	2.7	100	23.85
Civil registration coverage of cause of death (%)	116	83.35	0	100	24.82
Ill defined causes in cause of death registration (%)	91	12.75	1.68	46	9.55
*Essential medicines*^e^					
Median availability of selected generic medicines in public health facilities (2007–13)	39	56.13	0	100	28.69
Median availability of selected generic medicines in private health facilities	42	64.17	2.8	100	25.18
*Health financing*					
Current health expenditure (as % of GDP) (2017)^f^	181	6.63	1.2	17.1	2.85
Domestic general government health expenditure as % of GDP (2017)^g^	183	14	0.4	89.4	20.12
General government expenditure on health as a proportion of general government expenditure^h^	135	11.43	1.4	26.9	5.09
Out-of-pocket payments for health (% of current expenditure on health)^i^	184	31.22	0.1	80.95	19.22
*Global health security indicators (all scores out of 100)*^j^					
Prevention of emergence or release of pathogens	191	34.6	1.9	83.1	17.07
Early detection and reporting for epidemics of potential international concern	191	42.06	2.7	98.2	23.98
Rapid response to and mitigation of the spread of an epidemic	191	38.68	16	91.9	15.24
Sufficient and robust health system to treat the sick and protect health workers	191	26.67	0.3	73.8	17.27
Commitment to improving national capacity, financing and adherence to norms	191	47.97	23.3	85.3	12.76
Overall risk environment and country vulnerability to biological threats	191	54.65	6.2	87.9	17.14
Overall score- global health security	191	40.17	16.2	83.5	14.79
*Socio demographic factors*					
Female literacy rate	81	92.27	55	100	10.71
Proportion of people aged 60 years or more	104	30.56	0.18	100	28.55
Proportion of people below poverty line	87	19.96	0.06	61.9	12.02
*Governance*					
Voice and accountability	137	0.19	− 2.2	1.73	0.98
Political stability	139	0.12	− 3	1.61	0.98
Government effectiveness	137	0.21	− 2.24	2.23	0.99
Regulatory quality	137	0.18	− 2.34	2.21	1
Rule of law	137	0.2	− 2.33	2.05	1
Control of corruption	137	0.18	− 1.8	2.21	1.02

^a^https://www.who.int/workforcealliance/knowledge/resources/GHWA-a_universal_truth_report.pdf

^b^OECD library https://data.oecd.org/healthres/medical-graduates.htm

^c^https://apps.who.int/gho/data/node.main.HWFGRP_0020?lang=en

^d^https://apps.who.int/gho/data/node.main.HWFGRP_0040?lang=en

^e^https://apps.who.int/gho/data/node.main.488?lang=en

^f^https://apps.who.int/gho/data/node.main.GHEDCHEGDPSHA2011?lang=en

^g^https://apps.who.int/gho/data/node.main.GHEDGGHEDGDPSHA2011?lang=en

^h^https://apps.who.int/gho/data/node.main.GHEDGGHEDGGESHA2011?lang=en

^i^https://apps.who.int/gho/data/node.main.GHEDOOPSCHESHA2011?lang=en

^j^https://www.ghsindex.org/

The first part of the analysis assessed the correlation of health system variables with cumulative cases per million population. (Table 2) GHS indicators seemed to have a strong correlation with cases and deaths in bivariate analysis. However, when adjusted for other health systems factors, it did not necessarily translate into less burden of cases or deaths in the multivariable analysis. Correlational analysis of health system indicators with COVID-19 cases and deaths revealed a consistent association with health financing parameters.

**Table 2:** Correlation of health systems and GHS factors with cumulative cases and deaths per million population

Variable	Correlation coeff with Cumulative cases/million pop			Correlation coeff with Cumulative deaths/million pop	
	*n*	Spearman's rho	*P* value	Spearman's rho	*P* value
*Health service delivery*					
Number of inpatient beds per 10,000 population	143	0.3283	0.0001	0.2475	0.0029
*Health workforce*					
Number of health workers per 10,000 population	33	0.3657	0.0364	0.3565	0.0417
Annual number of graduates (doctors) of health professions educational institutions per 100,000 population	34	0.1264	0.4762	0.104	0.5583
Annual number of graduates (Nurses) of health professions educational institutions per 100,000 population	33	− 0.0985	0.5856	− 0.3128	0.0763
Doctor population ratio (per 10,000 pop)	165	0.5911	< 0.001	0.5465	< 0.001
Nursing and midwifery personnel (per 10,000 pop)	164	0.5373	< 0.001	0.4365	< 0.001
*Health information*					
Civil registration coverage of births (%)	152	0.5829	< 0.001	0.5175	< 0.001
Civil registration coverage of cause of death (%)	109	0.138	0.1526	0.1462	0.1292
Ill defined causes in cause of death registration (%)	90	0.1114	0.2958	0.0183	0.8643
*Essential medicines*					
Median availability of selected generic medicines in public health facilities (2007–13)	38	0.2757	0.0939	0.2084	0.2092
Median availability of selected generic medicines in private health facilities	41	0.2986	0.0579	0.2442	0.1238
*Health financing*					
Current health expenditure (as % of GDP) (2017)	162	0.3443	< 0.001	0.3984	< 0.001
Domestic general government health expenditure as % of GDP (2017)	163	0.2321	0.0029	0.3133	< 0.001
General government expenditure on health as a proportion of general government expenditure	123	0.4017	< 0.001	0.4384	< 0.001
Out-of-pocket payments for health (% of current expenditure on health)	164	− 0.2315	0.0029	− 0.1303	0.0963
*Global health security indicators (all scores out of 100)*					
Prevention of emergence or release of pathogens	170	0.4334	< 0.001	0.4344	0 < 0.001
Early detection and reporting for epidemics of potential international concern	170	0.3283	< 0.001	0.3513	< 0.001
Rapid response to and mitigation of the spread of an epidemic	170	0.3347	− < 0.001	0.3191	< 0.001
Sufficient and robust health system to treat the sick and protect health workers	170	0.4776	< 0.001	0.4471	< 0.001
Commitment to improving national capacity, financing and adherence to norms	170	0.1145	0.137	0.1504	0.0502
Overall risk environment and country vulnerability to biological threats	170	0.5826	< 0.001	0.4631	< 0.001
Overall score- global health security	170	0.4637	< 0.001	0.4424	< 0.001
*Socio demographic factors*					
Female literacy rate	76	0.29	0.0111	0.1695	0.1432
Proportion of people aged 60 years or more	98	− 0.2912	0.0036	− 0.4059	< 0.001
Proportion of people below poverty line	78	− 0.1085	0.3444	0.1069	0.3515
*Governance*					
Voice and accountability	125	0.279	0.0016	0.2485	0.0052
Political stability	126	0.1551	0.083	-0.049	0.5855
Government effectiveness	124	0.3359	0.0001	0.1833	0.0416
Regulatory quality	124	0.3977	< 0.001	0.2706	0.0024
Rule of law	124	0.32.3	0.0003	0.1277	0.1576
Control of corruption	124	0.2711	0.0023	0.0957	0.2903

Among all the factors studied, those predictive of cumulative cases per million population included infrastructural, human resource and financing parameters such as adequate inpatient beds health workers, expenditure on health as percent of GDP and also GHS parameters as prevention of emergence of pathogens and early detection and reporting of epidemics of international concern. Similarly, coverage of civil registry of births may indicate better reporting system and hence may suggest better use of data for taking timely actions. Achieving GHS parameters and numbers of doctors or health workers did not predict less numbers of cases. (Table 3).

**Table 3:** Multivariable analysis on health systems and GHS predictors of cumulative COVID-19 cases per million population

Variables	Regression coefficient (95% CI); *p* value
Number of inpatient beds per 10,000 population	− 118.39 (− 150.86, − 85.92); < 0.001
Number of health workers per 10,000 population	70.11 (10.43, 129.78); 0.025
Doctor population ratio (per 10,000 pop)	364.8 (297.88, 431.72); < 0.001
Overall score- global health security	569.16 (169.43, 968.89); 0.01
Civil registration coverage of births (%)	− 98.77 (− 162.02, − 35.53); 0.006
Prevention of emergence or release of pathogens	− 273.81 (− 464.31, − 83.32); 0.009
Domestic general government health expenditure as % of GDP (2017)	− 102.6 (− 163.4, − 41.79); 0.003
General government expenditure on health as a proportion of general government expenditure	340.66 (93.71, 587.61); 0.01
Early detection and reporting for epidemics of potential international concern	− 231.52 (− 378.88, − 84.17); < 0.001

The predictors of cumulative deaths per million population included general government expenditure on health as a proportion of general government expenditure, responsiveness of the system to prevent the emergence and release of pathogens and governance related voice and accountability. (Table 4) Interestingly, number of cases was weakly correlated with number of deaths. Voice and accountability as a positive predictor may suggest better reporting of deaths due to better governance.

**Table 4:** Multivariable analysis on health systems and GHS predictors of COVID-19 deaths per million population

Variables	–Regression coefficient (95% CI); *p* value
General government expenditure on health as a proportion of general government expenditure	− 24.56 (− 39.43, − 9.69); 0.003
Cumulative cases per 1 million population	0.01 (0.004, 0.019); 0.004
Prevention of emergence or release of pathogens	− 12.39 (− 20.85, − 3.93); 0.007
Voice and accountability	121.82 (42.11, 201.53); 0.005
Rapid response to and mitigation of the spread of an epidemic	17.39 (8.81, 25.97); 0.001
Domestic general government health expenditure as % of GDP (2017)	8.85 (5.53, 12.18); < 0.001

Proportion of population aged more than 60 years showed a strong association with COVID-19 cases and deaths in the unadjusted analysis but not when adjusted for health systems factors. Neither socio economic status reflected from the proportion of population below poverty line nor female literacy seemed to have any influence on the number of cases or deaths.

## Discussion

An ecological analysis of health systems indicators of 203 countries suggest that health financing parameters and preventive activities with regard to emergence of pathogens were better predictors of cumulative COVID-19 cases and deaths per million population compared to other health systems and GHS indicators.

A lot of scepticism surrounds the use of appropriate indicators and computation of health system indices [12]. Most of the criticisms focus on the technical aspects of constructing the composite index [13]. In several instances, validation exercises are conducted across countries before they are put to use. Health systems data utilize different approaches to collecting, systematising and communicating knowledge. Self reporting by Government yield data that are often biased and inaccurate. Such assessments are likely to give rise to erroneous results. For instance, the GHS data relies on wide-ranging sources of data to feed into a uniform classification system that ranks different countries [14]. Studies that have assessed the preparedness to prevent, detect and respond to infectious disease threats showed that GHS is not predictive [5–9]. Most of them analyzed GHS in isolation to assess correlation with numbers of COVID cases and deaths [5–8], except one that was more holistic, yet was restricted to African countries only [9]. Our study also echoes that GHS is not predictive when adjusted for a range of health systems parameters.

Health systems performance is presumably the key determinant that should be held accountable for any public health crisis. Capacity of health systems is usually assessed according to its structure, the health expenditure concerning the GDP, some indicators of development of a country, as stand alone components or in combination. Increasing resources for national health system are critical to improving health in developing countries. However, using existing resources more efficiently is accorded more importance than their mere availability. This is evident from a lack of appreciable differences observed between different health systems models such as the Beveridge model (that provides health care for all citizens and is financed by the government through tax payments), the Bismarck model (that uses an insurance system and is usually financed jointly by employers and employees through payroll deduction), the National Health Insurance (NHI) model (that has elements of both the Beveridge and Bismarck models—it uses private-sector providers, but payment comes from a government-run insurance program that all citizens fund through a premium or tax), and the “Out-of-Pocket” model [15].

Universal health coverage (UHC) aims to provide access to essential health services to all population and provide protection against catastrophic health spending. A robust health financing system is the cornerstone to achieve UHC [16]. General government expenditure on health as a proportion of general government expenditure is a core indicator of health financing systems. This indicator contributes to understand the weight of public spending on health within the total value of public sector operations [10]. It includes not just the resources channelled through government budgets but also the expenditure on health by parastatals, extrabudgetary entities and notably the compulsory health insurance. It refers to resources collected and pooled by public agencies including all the revenue modalities.

Although per capita health spending has significantly increased worldwide, such gains are varied, and most increases in health spending and pooled health spending are concentrated among upper-middle-income and high-income countries rather than lower- middle-income and low-income countries [16]. Ensuring that all countries have sustainable pooled health resources is crucial to the achievement of UHC [16]. Previous studies have explored how to translate health resources into achieving UHC by offering cost estimates for UHC attainment. Although such studies can be useful for initial planning purposes, they often fail to account for system inefficiencies and implementation challenges associated with programmatic scale-up. Moreover, measures of health-service costs are fundamentally different from quantification of the amount of total health spending needed to implement and sustain national health systems [16]. Countries that have closely aligned polices of UHC and GHS have fared relatively better [17].

Dissemination of information regarding performance of hospitals or providers, is important in ensuring the transparency and accountability of government spending and decision-making [18]. It is important to focus on effective coverage for better understanding of benefits accrued from UHC [19]. The area of health financing calls for more research and understanding in order to mount an effective public health response during crises [20].

The pandemic has exerted enormous pressure on health systems around the world highlighting the sub optimal resilience of countries, even those that were labelled as strong by various assessments. This builds up a case for rethinking on the metrics that would be more reflective of the ground situation and would be more resilience relevant [2]. Resilience should embark upon the principles of absorptive (ensuring stability because it aims to prevent or limit the negative impact of any disaster), adaptive (general ability of institutions, systems, and individuals to adjust to potential damage, to take advantage of opportunities, or to cope with the consequences) and transformative (system's ability to transform itself in response to changing conditions and disruptions) capacities [12]. Use of indicators that may not be most appropriate have masked the real situation and probably have instilled a false sense of confidence to face such global threats.

A responsive health system is expected to be well prepared to address health emergencies without being overwhelmed by the load of new cases. While resilience is a core concept in disaster risk reduction, its application to health systems is relatively new. It is high time that we start investigating not only on preparedness but on health systems strengthening [21]. Health-systems resilience, reflects an ability to adapt and respond and is, therefore, critical to assess [17]. A remodelled global health framework that envisages integration of UHC and GHS policies and approach, coupled with innovative and unified health financing, cross-sector resilience, equity and reliable indicators to measure those, offer an opportunity to strengthen health systems performance [17].

The analysis suffers from limitations in terms of missing data for certain variables from many countries. We have analyzed the health systems variables of countries with and without missing data. Descriptive analysis showed that more than 90% of the variables were similar between the two categories suggesting thereby that missing data might not have impacted the analyses significantly. Indicators that were readily available for each health systems block was considered, most of which seemed to be proxy indicators. Also, the time frame for the latest data considered for this analysis varied between countries. However, we do not anticipate any gross deviation in country specific data so as to influence the results significantly. There are concerns around reporting of actual numbers of COVID-19 cases and deaths from many countries. The actual numbers of cases and deaths are likely to be grossly underestimated. Health workforce and access to medicines could be critical predictors but could not be explored due to missing data. Despite these limitations, the study has an advantage of providing a comprehensive analysis of health systems factors across the world. It goes beyond the documented evidence on analysis of GHS index in isolation, only to assess correlation. It gives an insight into critical factors that are predictive of number of deaths—the ultimate measure of health systems resilience that includes governance, health care workforce, health care financing, availability of medicines and many more. The analysis will benefit key stakeholders and initiatives that are aligned across GHS and UHC such as multilateral organizations, national governments, International Health Regulations and many more. The study echoes the viewpoints of several reports, in diverse contexts, that suggest that more robust and valid indicators are required to assess the performance of health systems, those that will truly guide the countries to be better prepared for any crises that may come up in future.

## Conclusions

To conclude, health systems resilience reflects that ability of any country to tackle public health crises, without letting the existing system overwhelmed. It requires deeper analysis beyond the GHS indicators for better prediction of health systems performance. More robust and valid indicators are therefore required to assess the performance of health system so as to guide countries better. A comprehensive analysis of health systems indicators spanning governance, health workforce, health service delivery, health care financing, availability of medicines and GHS suggest that health financing parameters are better predictors of cumulative COVID-19 morbidity and mortality compared to other health systems and global health security indicators.

## Data Availability

The study is based on analysis of secondary data available in public domain.

## Abbreviations

GHS: Global Health Security
CEST: Central European Summer Time
WHO: World Health Organisation
GDP: Gross domestic product
NHI: National Health Insurance
UHC: Universal health coverage
